# Supporting families to care for people with dementia

**DOI:** 10.2471/BLT.17.021117

**Published:** 2017-11-01

## Abstract

The burden of care for people with dementia in low- and middle-income countries falls mainly on their families. Vijay Shankar Balakrishnan and Fiona Fleck report on some of the training and support that is available.

While doing his master’s degree in preventive medicine in 1999, Dr Amit Dias had difficulty finding people with dementia in Goa, India, to take part in a study.

“When we asked people whether they knew someone with ‘dementia’, they didn’t understand,” says Dias, who is now an assistant professor in the Department of Preventive and Social Medicine at Goa Medical College.

“But when we said, ‘do you know an elderly person who is forgetting his or her way home or who gets lost in his or her community?’ people pointed out someone, and the numbers grew.”

In 2010, Dias co-authored the *Dementia India report *of the**Alzheimer’s and Related Disorders Society of India, in which they estimated the prevalence of dementia for the first time. That prevalence has since increased to 4.1 million in this country of 1.3 billion people. 

India, as well as Ghana and Lebanon are among the first developing countries to produce prevalence data on dementia that can be used to raise awareness about the problem and design a suitable response. 

People with dementia show progressive loss of memory, find everyday tasks increasingly difficult and may display aggression, apathy and other forms of challenging behaviour. Dementia is caused by several diseases affecting the brain, of which Alzheimer's disease (60–70% of cases) and vascular dementia (20-30%) are the most common.

Dementia is a major cause of disability and dependency and it can be overwhelming for people with dementia, as well as their carers and families. 

“Dementia is often mistakenly considered to be a normal part of the ageing process and may go unrecognized in many countries,” says Dr Anne Margriet Pot from the Ageing and Life Course Department at the World Health Organization (WHO) in Geneva.

There is no cure or treatment to halt the progression of this chronic disease and so care is focused on the management of symptoms. 

“Care for dementia ideally starts with early diagnosis, which allows for the optimal management of symptoms, as well as the identification of physical, behavioural and psychological symptoms,” Pot says, adding: “These symptoms need to be monitored over the years – and some of them can be treated.

“In many low- and middle-income countries, long-term care for people with dementia relies solely on families. That’s why it’s vital to provide family carers with information about dementia and to train them in caregiving skills,” she says.

An estimated 47 million people live with dementia worldwide – some 58% of them in low- and middle-income countries – and these numbers are projected to reach 131.5 million by 2050, according to the *World Alzheimer report* *2016*.

Despite rapid increases in its prevalence, many governments have yet to recognize dementia as a public health priority and to provide much-needed dementia care and support for those affected and their families. 

 “The huge majority of people with dementia have not received a diagnosis, and so are unable to access care and treatment,” said Glenn Rees, the chair of Alzheimer’s Disease International. 

“Even when dementia is diagnosed, the care provided is too often fragmented, uncoordinated, and unresponsive to the needs of people living with dementia, their carers and families. This is unacceptable,” Rees said. 

Dias developed and evaluated a home-based intervention for the families of people with dementia in Goa involving non-specialist health workers. A randomized controlled trial, published in the journal *PloS One* in June 2008, showed that the invention reduced the burden on the carers while improving the quality of life of the people with dementia.

Dias also coordinates a research group in India that campaigns to highlight the fact that only a fraction of research funds for dementia is invested in low- and middle-income countries, where most people with dementia live.

“Caring for people with dementia is often stressful and can lead to family conflicts and burnout. Sometimes women leave their husbands because they cannot cope with the additional responsibility of looking after their mothers-in-law with dementia,” Dias says. 

"Often all the adults in the family are working, and so while they are at work they have to lock their parents with dementia in their homes to keep them safe.”

“Caring for people with dementia is often stressful and can lead to family conflicts and burnout.”

Amit Dias

Seven years ago, a 72-year-old woman with dementia called Ama Ahima was tortured and burned to death in Ghana by a group of people who accused her of being a witch. The woman’s grieving son explained afterwards that his mother had left home to visit him and lost her way.

The case of Ahima made headlines in Ghana and around the world, and came to symbolize the lack of awareness of dementia as well as the stigmatization and abuse of people with mental health conditions. 

While travelling to Scotland to do a master’s degree in nursing, Esther Dey read about Ahima and was so struck by what happened to her that she switched her studies to a diploma in dementia care instead.

“If those people had been aware of her condition, I don’t think they would have accused her of witchcraft,” says Dey, who returned to Ghana in 2012 to establish Alzheimer’s Ghana with NGO Alzheimer’s International.

Dey and her colleagues work to dispel the stigma attached to dementia, as well as the misperceptions, by collaborating with the media to raise awareness, and giving workshops and lectures to people from all walks of life, from families and church groups to students.

“We provide training and support for dementia carers. Raising awareness is important because without this, people with dementia may be deprived of the care they need and their families may not receive training or support to look after them,” Dey says.

In 2013, Ghana launched its national ageing policy recognizing the need for better care services for older people. “Our national policy is a good start. It’s been difficult to convince the government that more needs to be done about dementia in our country, now they are starting to understand,” Dey says. 

In India, Dias and his colleagues from NGO Sangath also train family members to identify and give care to people with dementia. 

This training includes some basic education about the disease, including the behavioural problems, as well as training on how to care for people with dementia at home, when to refer the person to a doctor and where to get help.

Monique Chaaya, a professor at the Department of Epidemiology and Population Health at the American University of Beirut in Lebanon, agrees that support for families caring for people with dementia should be given higher priority.

Chaaya speaks from her own personal experience: her aunt has been living with dementia for several years and is cared for by her husband.

“Over time, it has become so physically demanding and challenging for my uncle that his care-giving role affected his own mental health and he started to suffer from bouts of anger,” Chaaya says.

She and her colleagues published the first estimate of the prevalence of dementia in Lebanon in the journal *Alzheimer’s and dementia* in June. They found that 9% of people aged over 65 years in Lebanon were living with dementia and that most of them were cared for by their families.

Family care in Lebanon is common, Chaaya says, partly because of the limited availability of places in nursing homes and partly because people fear abuse of older people in such homes, but mainly because caring for ageing relatives is part of the culture.

“Collective care – such as the care farms in the Netherlands and other high-income countries – could help patients and relieve their care-givers in Lebanon, but given our family-oriented culture the emphasis should be on supporting carers at home,” Chaaya says. 

WHO’s Member States adopted the first global plan on dementia in May of this year. The *Global action plan on the public health response to dementia* 2017–2025 calls for better care and support for people with dementia and their carers.

Earlier this year WHO started piloting iSupport, an online pilot training programme for carers of people living with dementia, and is inviting feedback on it from Member States. 

The programme provides basic information for carers about the disease and how to deal with difficult behaviour, as well as how carers can – and should – take care of themselves as well. 

Last month WHO released *Integrated care for older people: guidelines on community-level interventions to manage declines in intrinsic capacity,* which provides advice on how public health authorities can better respond to the dementia challenge.

“There is an urgent need to develop sustainable, equitable and effective long-term care systems that meet the needs of people living with dementia and their carers across the course of the illness,” Pot says.

“Long-term care – even that provided free by family members – always has a cost. A core policy issue is how these costs can be equitably shared across societies.”

Anne Margriet Pot

“Long-term care – including that provided free by family members – always has a cost. A core policy issue is how these costs can be equitably shared across societies.”

“Only an integrated long-term care system, adapted to local settings, culture and resources, would work to enable people with dementia to continue to live lives of meaning and dignity regardless of their disease.”

**Figure:**
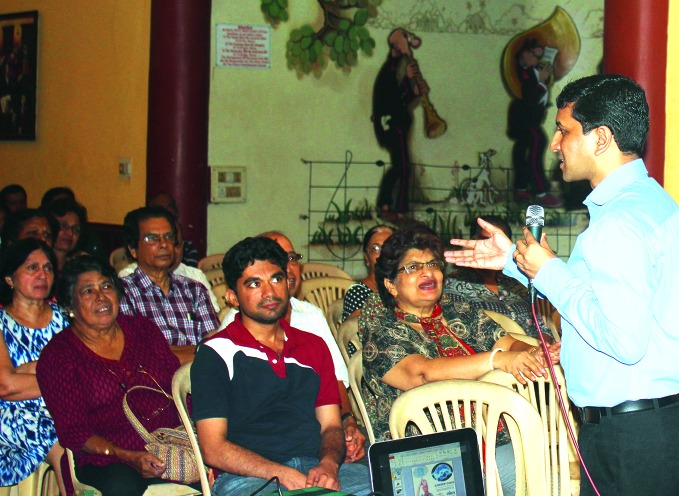
Amit Dias gives a workshop for families in India.

Courtesy of Amit Dias

**Figure:**
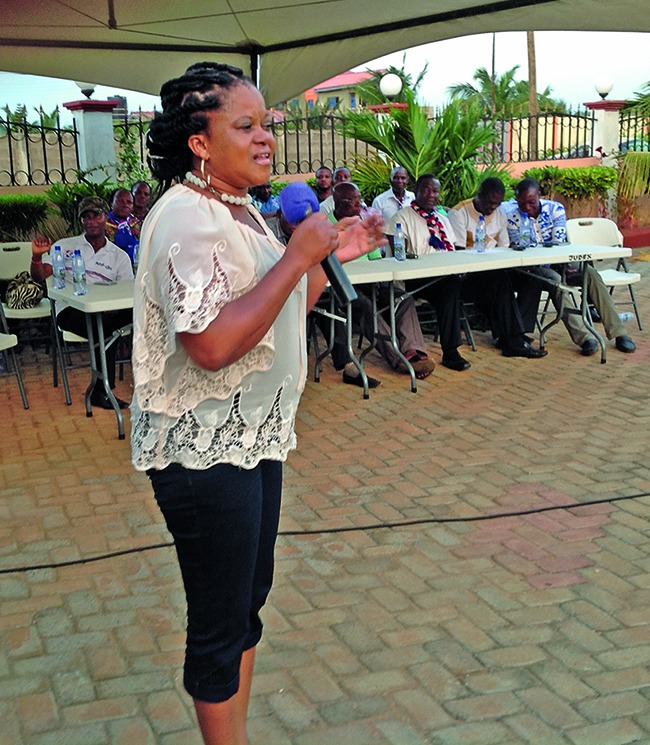
Esther Dey talks to a faith-based group in the city of Tema in Ghana.

Alzheimer’s Ghana

